# Editorial: Temporal lobe dysfunction in neuropsychiatric disorder

**DOI:** 10.3389/fpsyt.2022.1077398

**Published:** 2022-11-07

**Authors:** Yujun Gao, Qinji Su, Liang Liang, Haohao Yan, Fengyu Zhang

**Affiliations:** ^1^Department of Psychiatry, Renmin Hospital, Wuhan University, Wuhan, China; ^2^Department of Psychiatry, The Second Affiliated Hospital, Guangxi Medical University, Nanning, China; ^3^Department of Psychology, The Fourth Affiliated Hospital, Xinjiang Medical University, Urumqi, China; ^4^Department of Psychiatry, The Second Xiangya Hospital, Central South University, Changsha, China; ^5^Global Clinical and Translational Research Institute, Bethesda, MD, United States

**Keywords:** temporal lobe dysfunction, neuropsychiatric disorder, functional connectivity, temporal lobe epilepsy (TLE), major depressive disorder, schizophrenia, temporal lobe (TL), resting-state functional MRI

The temporal lobe is a significant part of the human brain forming the cerebral cortex with other lobes. It spans several cortical regions paralleling five temporal gyri, including the superior temporal gyrus (STG), the middle temporal gyrus (MTG), and inferior temporal gyrus (ITG), the fusiform gyrus, and the parahippocampal gyrus/entorhinal gyrus (1). The temporal lobe directly interacts with the limbic system, which plays essential roles in cognitive systems, emotional regulation, autonomic nervous systems, and information relay. Limbic circuits are involved in multiple neuropsychiatric disorders (2). However, the underlying neural mechanisms remain to be elucidated.

This Research Topic published 21 articles that include 20 original research using resting-state fMRI (rs-fMRI) in patients with neuropsychiatric and related disorders relative to healthy controls (HCs). Seven studies focused on temporal lobe epilepsy (TLE); Five on major depressive disorder (MDD), two of which patients underwent antidepressant treatment; Two studies about schizophrenia (SCZ); Others focused on monocular blindness (MCB), congenital blindness (CB), primary nocturnal enuresis (PNE), acoustic neuroma, sudden sensorineural hearing loss (SSHL), premenstrual syndrome (PMS), and premenstrual dysphoric disorder (PMDD).

## Temporal lobe epilepsy

Two studies provided consistent evidence that patients with TLE had decreased FC in the bilateral MTG. Based on the voxel-mirrored homotopic connectivity (VMHC) (3), Wu et al. found that patients with lTLE exhibited a decreased VMHC in the bilateral MTG and middle cingulum gyrus (CG). With a similar study design, Chu et al. found that patients with the right TLE (rTLE) had a decreased VMHC in the bilateral superior temporal pole (STP), middle temporal pole (MTP), MTG, ITG, and inferior frontal gyrus (IFG). In contrast, they exhibited an increase of VMHC in the bilateral precentral gyrus (PreCG), the postcentral gyrus (PoCG), and the supplemental motor area (SMA).

Patients with TLE may have altered network connectivity. Li et al. studied network homogeneity (NH) of the ventral somatomotor network (VSN) in patients of lTLE and rLTE and found reduced NH in the bilateral Rolandic operculum and STG, but increased in PoCG. Huang et al. showed rTLE patients had a decreased NH of default mode network (DMN) in the R-ITG and L-MTG, but an increase in the bilateral PCu and R-inferior parietal lobe. Based on the degree of centrality (DC) (4), Guo et al. showed that patients with rTLE had a reduced DC in the R-caudate, but increased network connectivity in the L-MTG, superior parietal gyrus (SPG), inferior parietal gyrus (IPG), superior frontal gyrus (SFG), medial SFG, IFG, and R-precuneus (PCu).

The basal forebrain (BF) is a cluster of subcortical structures providing axonal projection to the entire cerebral cortex and comprises four nuclei of the medial septal (MS, Ch1), the vertical limb of the diagonal band of Broca (vDbB, Ch2), the horizontal limb of the diagonal band of Broca (hDbB, Ch3), and the nucleus basalis of Meynert (nBM, Ch4) (5). Fan et al. studied TLE patients with and without focal to bilateral tonic-clonic seizure (FBTCS), using four basal forebrain subregions to examine the resting-state FC (rsFC). Regardless of FBTCS status, the TLE patients had reduced rsFC of BF subregions with the cerebellum, striatum, DMN, frontal and occipital lobes. Decreased rsFC of Ch1-3 with bilateral striatum and left cerebellum posterior lobe and Ch4 with bilateral amygdala was associated with FBTCS.

## Major depressive disorder

MD is highly heterogeneous and associated with multi-faceted risk factors (6, 7). While neuroimaging studies have shown that patients with MDD tend to have altered brain structure and FC (8, 9), there is significant inconsistency. Five studies focused on brain activity and functional connectivity in MDD using various neuroimaging techniques (Supplementary Box).

Two studies examined brain activities in patients with MDD relative to HCs and after antidepressant treatment. Wang et al. studied drug-naïve first episode of adolescent MDD who also had 2-week electroconvulsive therapy (ECT). Whole brain voxel analysis showed increased activity in the R-orbital IFG, inferior occipital gyrus (IOG), and L-middle frontal gyrus (MFG) in patients with MDD. Meanwhile, patients treated with ECT significantly increased brain activity in the R-medial SFG, anterior cingulate gyrus (ACG), paracingulate gyrus (paraCG), medial CG and PCG, dorsolateral SFG, and L-MFG. Also, the increased brain activity in the frontal gyri seemed to be significantly associated with reducing clinical severity. Xiong et al. detected a high activity in the bilateral MFG, CG, and MTG in MDD; patients treated with vortioxetine for 2 weeks had decreased brain activity in the R-ITG, but increased activity in the L-low cerebellum, R-CG, and central posterior gyrus.

Three studies focused on regional homogeneity and network connectivity. Song et al. found that patients with drug-naïve first-episode MDD had an increase of ReHo in the L-anterior cingulate cortex (ACC) but decreased ReHo in the L-PreCG. However, none of these were significantly associated with the HAMD score. Luo et al. studied treatment-naïve first-episode MDD and HCs matched by gender, age, and education. They found a decreased NH in the R-PCu and abnormal executive control reaction time relative to HCs; the decreased NH was not significantly correlated to the clinical severity. In addition, Lin et al. conducted a larger-size study of 198 cases with MDD and 234 HCs using network centrality measure. Patients with MDD exhibited an elevated level of DC in the L-anterior cerebellar lobe, vermis, L-hippocampus, L-caudate, but a reduced DC in the L-posterior cerebellar lobe, L-insula, and R-caudate.

Han et al. conducted a clinical study of patients with MDD. They found that the frequency of negative evaluation and emotional words were significantly associated with the severity of MDD measured by HAMD-17.

## Schizophrenia

Activation of auditory-related brain regions is one of the neuropathological mechanisms in SCZ. Disruption of functional connectivity in the L-temporal lobe, particularly L-STG has been indicated in AVH (10). Xue et al. studied voxel-wised dynamic FC (dFC) of the primary auditory cortex—Heschl's gyrus (HES) and auditory association cortex (AAC) in drug-naïve first episode SCZ with and without AVH. They found SCZ patients with AVH had an increased dFC of L-AAC with R-MTG and middle occipital gyrus (MOG) but a decreased dFC of L-HES gyrus with L-superior CG, L-cuneus, and L-PCu gyri, and R-HES gyrus with posterior CG.

Inter-hemispheric disconnection has been a notable pathological finding in SCZ (11, 12). Chen et al. studied inter-hemispheric connectivity, focusing on the STG cluster (extending into Heschl's gyrus, insula, and Rolandic operculum) and fusiform cluster (growing into the para-hippocampus). SCZ with AVH showed a reduced VMHC in the fusiform cluster; A decreased VMHC in the STG cluster was observed in both SCZ with and without AVH compared to HCs.

## Acoustic neuromas and sudden sensorineural hearing loss

Acoustic neuroma (AN) is a type of tumor that develops from the sheath of Schwann cells and grows in the ear and can affect hearing and body balance function. While often described as transient, the psychiatric symptoms in patients with ANs may include mood change, memory loss, hallucination, and delusion (13). Deng et al. performed a clinical neuropsychological study of patients with ANs, and then an rs-fMRI investigation. Patients with ANs exhibited a cognitive decline, reduced ReHo, and decreased connectivity in the frontal lobe. Liu et al. found a reduced ReHo in the L-cerebellum, B-ITG, L-STP, R-para-hippocampal gyrus, L-PCC, and R-SFG in patients with SSHLcompared to HCs.

## Congenital and monocular blindness

Lack of visual experience may affect the development of brain structure and functions development. Hu J-J. et al. found that patients with CB decreased ReHo in the R-orbital MFG, bilateral MOG, and R-dorsolateral SFG, but an increase in the L-paracentral lobule, R-insula, and bilateral thalamus. Using the percentage of amplitude fluctuations (perAL), Hu Q. et al. found that patients with MCB had decreased brain activity in the middle OL and the L-middle cingulate lobe but increased activity in the frontal and temporal lobes.

## Meibomian gland dysfunction in severely obese population

Meibomian gland dysfunction is a chronic and diffuse abnormality of meibomian glands characterized by terminal duct obstruction. Xu et al. found that Meibomian gland dysfunction (MGD) in severely obese people exhibited decreased brain activity in the R-cerebellum, L-fusiform gyrus, R-medial orbitofrontal gyrus, L-triangle IFG, and L-IPG, but increased activity in the left lingual gyrus, R-globus pallidus, right ACG and para-CG, and L-MOG. The increased brain activity in R-cerebellum and left triangle IFG seemed to be associated with a high anxiety and depression score. Notably, this analysis was based on a tiny sample (*n* = 12).

## Primary nocturnal enuresis

The development of chidrlem with PNE may involve a brain-bladder control network. Zhong et al. performed a seed-based rsFC of PNE and HCs with the first attempt to focus on four regions of the left and right rostral hippocampus (rHipp) and caudal hippocampus (cHipp) as of interest (ROI). Children with PNE exhibited a decreased rsFC in L-rHipp with R-fusiform gyrus, R-Rolandic operculum, L-inferior parietal lobe (IPL), and R-PreCG, but a reduced rsFC in L-cHipp with R-fusiform gyrus and R-SMA. The reduced FC of L-rHipp with R-Rolandic operculum and left cHipp with fusiform was also associated with the disease duration. The altered functional connectivity in these brain regions may involve multiple brain networks, including the limbic system, sensorimotor, DMN, and frontoparietal network. In addition to the brain-bladder control network, these brain regions are important in cognitive function and emotion.

## Premenstrual syndrome and premenstrual dysphoric disorder

Premenstrual syndrome (PMS) is a group of clinically significant somatic and psychological symptoms or distress that women develop during the luteal phase of the menstrual cycle (about a week or two before the period) (14). Its severe form, premenstrual dysphoric disorder (PMDD), is classified as a subcategory of depression. Long et al. contributed a mini-review of neuroimaging studies of PMS and PMDD. Neuroimaging studies show more robust evidence of structural and functional alterations in the amygdala and the hippocampus's medial temporal lobe (MTL). Also, an fMRI study indicated abnormal fusiform gyrus activity in patients with PMS and PMDD.

## Summary

The studies indicated temporal lobe dysfunction in neuropsychiatric and related disorders. In TLE, decreased FC in the MTG, ITG, and Rolandic operculum were identified, and reduced FCs of BF subregions with the striatum and cerebellum were associated with both TLE and FBTCS. However, increased connectivity in the PreCG and PoCG was associated with TLE (Table 1). These findings consistently appear in at least one independent studies or samples within the same study. The reduced FC between the left rHipp and the right Rolandic operculum was associated with PNE. Moreover, AVH in SCZ might be related to increased dsFC in L-AAC, decreased connectivity in the HES gyrus, and reduced inter-hemispheric connectivity in the fusiform cluster.

**Table 1:** rs-fMRI study of temporal lobe dysfunctions in temporal lobe epilepsy, major depression, schizophrenia and other disorders.

**Study**	**Disease**	**Sample**	**Measure**	**Region of brain activity or connectivity**	
		**Cases vs. HCs**		**Decreased**	**Increased**
**Major depressive disorder**					
Wu et al.	L-TLE	59 vs. 60	VMHC	B-MTG, MCG.	
Chu et al.	R-TLE	58 vs. 60	VMHC	B-MTG, ITG, B-sup TP, B-middle TP, ITG, orbital IFG.	B-PreCG, PoCG, SMA
Li et al.	R-TLE	53 vs. 68	NH	B-Rolandic operculum, R- STG.	B-PoCG
	L-TLE	83 vs. 68	NH	R-Rolandic operculum.	L-PoCG
Huang et al.	R-TLE	42 vs. 43	NH	Left MTG, R-ITG.	B-PCu, R-inf parietal lobe.
Guo et al.	R-TLE	68 vs. 73	DC	R-caudate.	L-MTG, sup PreCG, SFG, IFG, IPG, med SFG, R-PCu.
Fan et al.	TLE and FBTCS vs. HCs	50 vs. 25	rsFC	BF with striatum, cerebellum, DMN, occipital lobe.	
	FBTCS vs. TLE	25 vs. 25	rsFC	BF with B-striatum, L-cerebellum poste lobe, B- amygdala.	
**Temporal lobe epilepsy**					
Wang et al.	Adolescent MDD	30 vs. 30	fALFF		R-orbital IFG, inf OG, L-MFG.
	ECT		fALFF		R-med SFG, dorsol SFG, R-MFG, ante CG, med CG, ParaCG.
Xiong et al.	MDD	25 vs. 25	ALFF		B-MFG, CG, and MTG
	Vortioxetine	25	ALFF	R-ITG	L-low cereb, R-CG, central poste gyrus.
Song et al.	MDD	52 vs. 45	ReHo	L-PreCG	L-ACC
Luo et al.	MDD	73 vs. 70	NH	R-PCu	
Lin et al.	MDD	198 vs. 234	NH	L-poste cerebellar lobe, L-insula, R- caudate.	L-anterior cerebellar lobe, Vermis, L-hipp, L-caudate.
**Schizophrenia**					
Xue et al.	SCZ AVH vs. NAVH+HC	107 vs. 85+104	dFC		L-AAC with R-MTG, middle OC.
			dFC	L-HES with L-SCG, L-cuneus gyrus, L-PCu gyrus.	
			dFC	R-HES with Poste CG.	
Chen et al.	• SCZ AVH vs. NAVH • AVH < NAVH < HC	• 42 vs. 26 • 42, 26, 82	• VMHC • VMHC	• Fusiform cluster (into paraHipp gyrus). • STG cluster (into Heschl's gyrus, insula, Rolandic operculum).	
**Other disorders**					
Deng et al.	Acoustic neuroma	64 vs. 67	ReHo	Frontal lobe	
Liu et al.	SSHL	27 vs. 27	ReHo	L-cerebellum, B-ITG, L-sup TL, R-paraHipp, L-poste CC, R-SFG.	
Hu et al.	Congenital blindness	23 vs. 23	ReHo	R-orbital MFG, B-middle OG, R-dorsol SFG.	L-paracentral lobule, R-insula, and B-thalamus
Hu et al.	Monocular blindness	29 vs. 29	PerAF	L-middle OL, R-middle OL, L-middle CL.	L-sup FL, L-inf orbital FL, L-inf TL, L-inf frontal operculum
Xu et al.	MGD in severe obese	12 vs. 12	fALFF	R-cereb, L-fusifom G, R-med orbito-FG, L-triangle IFG, L-IPG	R-globus pallidus, R-ante CC and para-CG, L-Middle OL.
Zhong et al.	Nocturnal euresis	30 vs. 29	rsFC	L-rHipp with R-FFG, G, R-Rolandic operculum, L-inf parietal lobe, R-PreCG; L-cHipp with R-fusiform G, R-SMA.	

(1) AVH, auditory verbal hallucination; ECT, electroconvulsive therapy; FBTCS, focal to bilateral tonic-clonic seizure; HC, Healthy control; L-TLE, left temporal lobe epilepsy; MDD, major depressive disorder; MGB, Meibomian gland dysfunction; R-TLE, right temporal lobe epilepsy; SSHL, sudden sensorineural hearing loss.

(2) DC, degree of centrality; fALFF, fractional amplitude of low-frequency fluctuations; NH, network homogeneity; perAF, percentage of amplitude of fluctuations; ReHo, regional homogeneity; rsFC, resting-state functional connectivity; VMHC, Voxel-mirrored homotopic connectivity.

(3) ACC, anterior cingulate cortex; BF, basal forebrain; CC, cingulate cortex; CG cingulate gyrus; CL, cingulate lobe; DMN, the default mode network; FFG, fusiform gyrus; FG, frontal gyrus; FL, frontal lobe; IFG, inferior frontal gyrus, ITG, inferior temporal gyrus; IPG, inferior parietal gyrus; MFG, middle frontal gyrus; MTG, middle temporal gyrus; OG, occipital gyrus; OL, occipital lobe; ParaCG, paracingulate gyrus; PCu, precuneus; PoCG, post-central gyrus; PreCG, precentral gyrus; SFG, superior frontal gyrus; SMA, supplementary motor area; STG, superior temporal gyrus; TG, temporal gyrus; TL, temporal lobe; TP, temporal lobe.

(4) Ante, anterior; B, bilateral; cereb, cerebellum; cHipp, caudal hippocampus; dorsol, dorsolateral; Inf, inferior; L, left; Med, medial; Poste, posterior; rHipp, rostral hippocampus; R, right. Sup, superior.

By contrast, findings from five studies of MDD seemed lacking consistency. Two studies with drug-naïve first episode MDD treated with antidepressant therapy did not find that antidepressant treatment significantly impacted the baseline brain activity associated with MDD. The inconsistent findings between disease association and response to treatment may pose a challenging question for interpretation. Part of the causes might be that patient heterogeneity had reduced the power to cause false-positive discovery.

The fMRI techniques have made it feasible to study the entire connectome at a large scale in humans. However, studies should move beyond the exploratory analysis of FC. Therefore, it calls for the rigor of study and advanced analytic techniques to maximize the critical tools of fMRI for neuroscience research. Furthermore, it might be more promising when fMRI combines with radionuclide scans such as positron emission tomography (PET) and structure MRI (sMRI), which can reveal the brain structure and functions at a circuit level. Multimodal neuroimaging may have a greater demand and challenge in visual data inspection, integration, and fusion for analysis (15).

## Author contributions

FZ reviewed all articles, summarized individual studies' findings, and drafted the manuscript. All other editors participated in editing articles and reviewed and had access to the manuscript and related Supplementary materials. All authors contributed to the article and approved the submitted version.

## Conflict of interest

The authors declare that the research was conducted in the absence of any commercial or financial relationships that could be construed as a potential conflict of interest.

## Publisher's note

All claims expressed in this article are solely those of the authors and do not necessarily represent those of their affiliated organizations, or those of the publisher, the editors and the reviewers. Any product that may be evaluated in this article, or claim that may be made by its manufacturer, is not guaranteed or endorsed by the publisher.
