# Supplementary material for: Editorial: Temporal lobe dysfunction in neuropsychiatric disorder
Source: Front Psychiatry. 2022 Nov 7;13:1077398. doi: 10.3389/fpsyt.2022.1077398 (PMC9677554; doi:10.3389/fpsyt.2022.1077398)
Supplement: Supplementary file 1 [file Data_Sheet_1.PDF]

Box: Abbreviations and terminology of neuroimaging methods

Abbreviation	Terminology	Definition and measurement
ALFF	The amplitude of low-frequency fluctuations	Measure spontaneous fluctuations in blood oxygen level-dependent (BOLD)-fMRI signal intensity in a given brain region, reflecting local brain activity
fALFF	The fractional amplitude of low-frequency fluctuation	Ratio of each frequency (0.01-0.08 Hz) to the entire frequency range (0-0.25 Hz), which improves the sensitivity and specificity in detecting the spontaneous brain activity
ICA	Independent component analysis	Assess rest-state brain network, but no clear consensus to compare components across subjects or groups.
ROI	Region of interest seed-based correlation	Assess the resting-state brain network, but sometimes it is hard to choose an appropriate ROI.
ReHo	Regional homogeneity,	Measure the local functional connectivity of a fixed node and the nearest neighboring nodes in the entire connectome and quantify the connection.
NH	Network homogeneity	Measure the mean correlation of a specific voxel's time series with the time series of every other voxel within the network, which provides an unbiased survey of an entire network without prior knowledge.
DC	Degree of centrality	Measure the number or strength of connection of a specific voxel's time series with the time series of every other voxel within the entire connectome in the whole brain
